# A Constant Light-Genetic Screen Identifies KISMET as a Regulator of Circadian Photoresponses

**DOI:** 10.1371/journal.pgen.1000787

**Published:** 2009-12-24

**Authors:** Raphaëlle Dubruille, Alejandro Murad, Michael Rosbash, Patrick Emery

**Affiliations:** 1 Department of Neurobiology, University of Massachusetts Medical School, Worcester, Massachusetts, United States of America; 2 Program in Neuroscience, University of Massachusetts Medical School, Worcester, Massachusetts, United States of America; 3 Howard Hughes Medical Institute, National Center for Behavioral Genetics and Department of Biology, Waltham, Massachusetts, United States of America; University of Texas Southwestern Medical Center, Howard Hughes Medical Institute, United States of America

## Abstract

Circadian pacemakers are essential to synchronize animal physiology and behavior with the day∶night cycle. They are self-sustained, but the phase of their oscillations is determined by environmental cues, particularly light intensity and temperature cycles. In *Drosophila*, light is primarily detected by a dedicated blue-light photoreceptor: CRYPTOCHROME (CRY). Upon light activation, CRY binds to the pacemaker protein TIMELESS (TIM) and triggers its proteasomal degradation, thus resetting the circadian pacemaker. To understand further the CRY input pathway, we conducted a misexpression screen under constant light based on the observation that flies with a disruption in the CRY input pathway remain robustly rhythmic instead of becoming behaviorally arrhythmic. We report the identification of more than 20 potential regulators of CRY-dependent light responses. We demonstrate that one of them, the chromatin-remodeling enzyme KISMET (KIS), is necessary for normal circadian photoresponses, but does not affect the circadian pacemaker. KIS genetically interacts with CRY and functions in PDF-negative circadian neurons, which play an important role in circadian light responses. It also affects daily CRY-dependent TIM oscillations in a peripheral tissue: the eyes. We therefore conclude that KIS is a key transcriptional regulator of genes that function in the CRY signaling cascade, and thus it plays an important role in the synchronization of circadian rhythms with the day∶night cycle.

## Author Summary

In most organisms, intracellular molecular pacemakers called circadian clocks coordinate metabolic, physiological, and behavioral processes during the course of the day. For example, they determine when animals are active or resting. Circadian clocks are self-sustained oscillators, but their free-running period does not exactly match day length. Thus, they have to be reset by environmental inputs to stay properly phased with the day∶night cycle. The fruit fly *Drosophila melanogaster* relies primarily on CRYPTOCHROME (CRY)—a cell-autonomous blue-light photoreceptor—to synchronize its circadian clocks with the light∶dark cycle. With a genetic screen, we identified over 20 candidate genes that might regulate CRY function. *kismet* (*kis*) is among them: it encodes a chromatin remodeling factor essential for the development of *Drosophila*. We show that, in adult flies, KIS is expressed and functions in brain neurons that control daily behavioral rhythms. KIS determines how *Drosophila* circadian behavior responds to light, but not its free-running period. Moreover, manipulating simultaneously *kis* and *cry* activity demonstrates that these two genes interact to control molecular and behavioral circadian photoresponses. Our work therefore reveals that KIS regulates CRY signaling and thus determines how circadian clocks respond to light input.

## Introduction

The rotation of the Earth on its axis is responsible for the mild temperatures found in most regions of the globe, which allow for a complex biosphere to thrive. However, this rotation is accompanied by important variations in light intensity and temperature, which are challenges for most organisms. Since the day∶night cycle has a stable period, the physical and ecological changes it induces in the environment can be temporally predicted. This anticipation is made possible in most organisms by circadian clocks. In *Drosophila*, the molecular circadian pacemaker is a transcriptional feedback loop: two proteins - PERIOD (PER) and TIMELESS (TIM) - repress their own gene transcription by interfering with the activity of the transcription factors CLOCK (CLK) and CYCLE (CYC) [1]–[4]. PER and TIM stability, their translocation into the nucleus, and their repressive activity are tightly regulated by kinases (DBT, CKII, and SGG) and phosphatases (PP2A and PP1) [5]–[14]. Importantly, while this molecular clock free-runs - i.e. its oscillations persist in absence of environmental cues - its period only approximates 24 hr and must thus be reset every day to be in phase with the day∶night cycle. This is the role of light and temperature input pathways.

The blue-light photoreceptor CRYPTOCHROME (CRY) is the main *Drosophila* circadian photoreceptor [15]–[17]. After absorbing a photon, CRY undergoes a conformational change involving its C-terminal domain and binds to TIM [18],[19]. TIM is then tagged for ubiquitination and proteasomal degradation [20],[21]. The mechanisms by which CRY initiates the cascade of events that leads to TIM degradation remain unclear. However, JETLAG (JET) plays an important role in targeting TIM for proteasomal degradation [22],[23]. JET is part of an SCF E3 ubiquitin ligase complex responsible for TIM ubiquitination. Interestingly, JET also regulates CRY's own light-dependent degradation [24]. The COP9 signalosome subunits CSN4 and CSN5 are also required for circadian behavioral photoresponses [25]. The CSN complex regulates the activity of SCF E3 ubiquitin ligases and might thus be functioning upstream of JET in circadian neurons. Another protein known to regulate the CRY input pathway is the kinase SGG [26]. SGG interacts with CRY, and its overexpression inhibits CRY activity, through as yet unclear mechanisms. There is also little known about the regulation of the expression and stability of the proteins involved in CRY photoreception. This is an important question, because the level of expression of these proteins needs to be tightly regulated so that circadian rhythms are tuned to the proper range of light intensities. They should be able to respond to subtle and progressive changes in light intensities at dawn or dusk, without being excessively sensitive to light and for example inappropriately respond to moonlight levels of illumination.

We therefore undertook a misexpression screen, which identified more than 20 genes that might affect circadian photoreception. We focused on one gene in particular, which encodes the chromatin remodeling protein KISMET. Indeed, by downregulating its expression with RNA interference, we found that KISMET is essential for CRY-dependent light responses.

## Results

### A circadian misexpression screen under constant light

**Figure 1:**
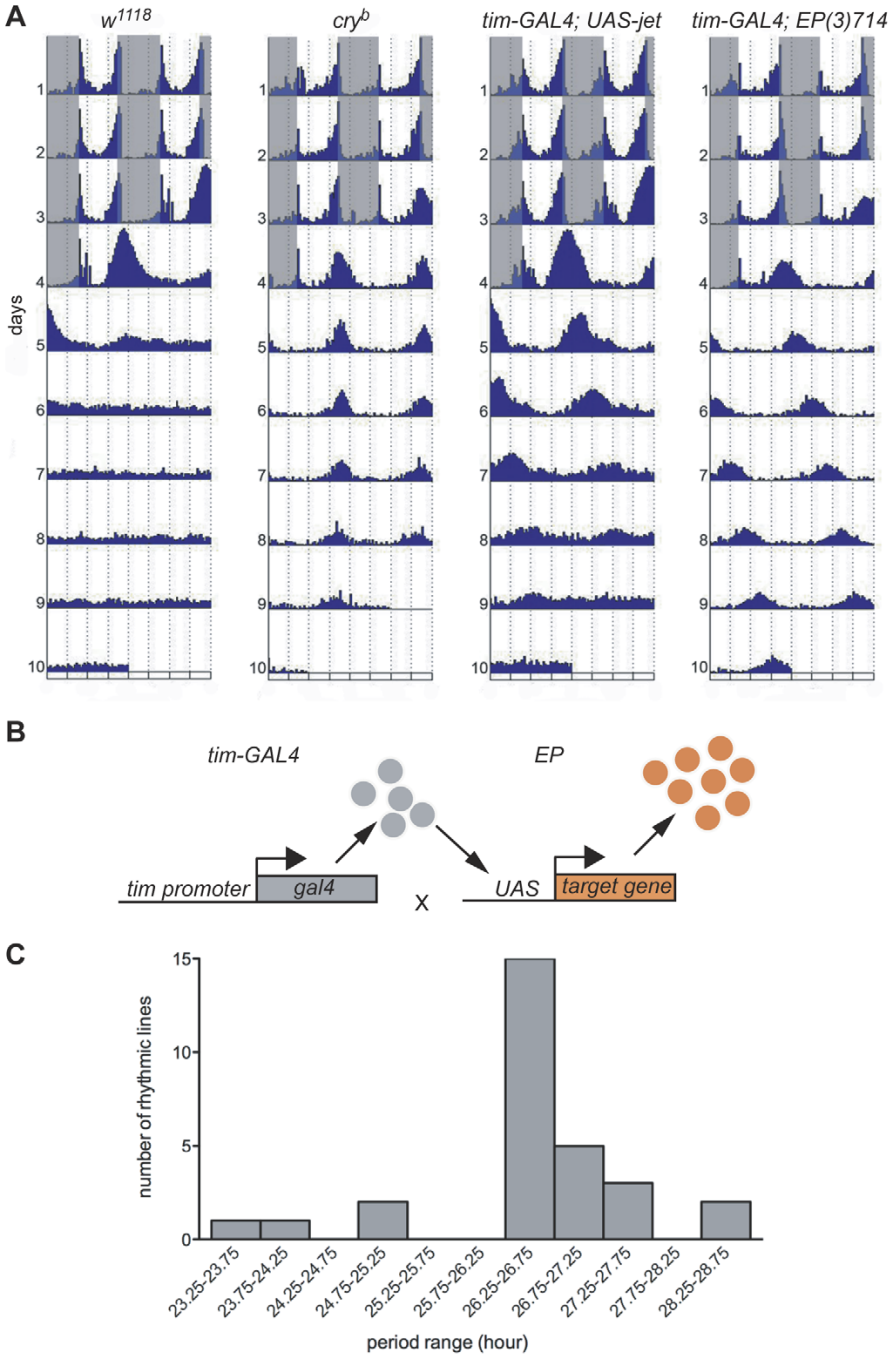
The misexpression screen in constant light (LL). (A) Average double-plotted actograms of control (*w^1118^*), *y w; cry^b^* (*cry^b^*), *jet* overexpressing (*y w*; *tim-GAL4;UAS-jet*) and *miR-282* overexpressing flies (*y w*; *tim-GAL4;EP(3)714*). Flies were entrained for 3 days in a 12hr∶12hr light∶dark cycle (gray shadings indicate the dark phase) and then released in constant light (LL). While control flies become arrhythmic in LL, *cry^b^* flies and *jet* and *miR-282* overexpressing flies remain rhythmic. All genotypes are homozygous for the *ls-tim* variant [22],[86],[87] (see also Materials and Methods). n = 15–16 flies/genotype. (B) The strategy of the misexpression screen. Each *EP* line bearing a pseudo-randomly inserted *P*-element containing UAS binding sites was crossed to *tim-GAL4* flies. Males from the progeny were tested for rhythmicity in LL. (C) Period range of the rhythmic *EP* lines (when crossed to *tim-GAL4*) in LL. Histograms show the number of rhythmic lines for each period range between 23.25 hr and 28.75 hr.

The circadian behavior of wild-type flies is dramatically affected by the presence of constant light (LL) at an intensity of 10 lux or more [27]. They become totally arrhythmic, while under constant darkness they would remain rhythmic for weeks. This circadian response to constant light is dependent on the circadian photoreceptor CRY. *cry^b^* flies, which carry a severely hypomorphic *cry* mutation (a quasi-null mutation), remain behaviorally rhythmic under constant light, with a periodicity of 24 hours, as if they were under constant darkness [15] (Figure 1A). To identify new components of the CRY light input pathway, we decided to screen the P. Rørth collection of *EP* lines under LL. These *EP* lines carry randomly inserted *P*-elements in their genome (Figure 1B) [28], which contain UAS binding sites. By crossing these *EP* lines to flies expressing GAL4 under the control of the *timeless* promoter (*tim-GAL4* flies), we overexpressed or in rare cases downregulated [28] the genes targeted by the *EP* element specifically in tissues with circadian clocks. Whether the targeted genes were up- or down-regulated depended on the orientation of the *EP* element insertion. A sense RNA is usually produced, which results in overexpression of the targeted gene (Figure 1B). Sometimes however, the *EP* element generates an antisense RNA. Thus, at least four mechanisms could explain how *EP* lines might be rhythmic in LL when crossed to *tim-GAL4*. First, we might overexpress a negative regulator of the CRY input pathway, such as SGG. Indeed, when SGG is overexpressed, flies are robustly rhythmic in LL [26]. Second, we might downregulate, with the few *EP* lines that generate antisense RNAs, genes crucial to CRY signaling. Third, the overexpression of a gene crucial to CRY signaling might be toxic for the CRY input pathway. Fourth, we might affect PER regulation, since its overexpression results in LL rhythms [26],[29]. Supporting our strategy, we found that overexpressing JET - which promotes light-dependent TIM degradation and hence circadian photoresponses [22],[23] - also results in rhythmic behavior in LL (Figure 1A), although the long period length we observed indicates that these flies are not entirely blind to constant light [27],[29].

We screened ∼1800 *EP* lines located on the second and third chromosomes, which target ∼1350 genes [30] or about 10% of the genome. 30 of these lines showed consistent rhythms in LL (>50% rhythmic flies, Table 1). This is a hit rate of 1.7%, which is in the range of other misexpression screens [28],[30]. In constant darkness (DD), none of these lines showed any obvious defect (Table S1). They were all robustly rhythmic, with a period length close to that of control flies. In LL however, the period length was usually not that of control flies in DD, or of *cry^b^* flies in LL. Periods were in most cases long, with most lines (20/30) showing a period range that was centered around 26.5–27 hr (Figure 1A and 1C and Table 1). Power was also weaker than under DD conditions, and the variability of period within lines was higher. This shows that the robustness of circadian rhythmicity is affected; this is not surprising since LL is disruptive to circadian rhythms. In sum, the selected lines are clearly not insensitive to LL, while *cry^b^* are virtually blind to constant light [15],[31]. Of note is that 10 lines did not fall into the 26.5–27 hr range. 3 lines with a very long period (27.5–28.5 hr) affected the same gene, *miR-282*. However and as mentioned above, these lines all showed normal period length in DD. Thus, these differences in LL period length are not due to pacemaker dysfunction, i.e., overexpression of specific genes results in specific LL phenotypes.

**Table 1:** Behavior of the selected *EP* lines crossed to *tim-GAL4* under constant light (1,000 lux).

Genotype (each *EP* lines is crossed to *tim-GAL4*)	Gene predicted to be affected	n	% of rhythmic flies	Period average (±SD)	Power average (±SD)
*EP(2)2367*	*morgue*	30	87	26.4±0.6	37.5±22.8
*EP(3)714*	*miR-282*	43	83	27.5±0.7	54.8±26.5
*EP(3)3718*	*miR-282*	31	78	27.5±1	37.8±23
*EP(2)670*	*GstS1*	33	73	26.5±1	49.1±23.2
*EP(3)703*	*cg8165/8176*	74	71	26.6±1.8	32.5±17.9
*EP(3)3041*	*miR-282*	33	70	28.5±1.1	37.3±21.3
*EP(2)965*	*elB*	49	67	27.7±2	31.3±20.5
*EP(3)972*	*calpB*	31	65	26.5±1.2	33.3±15.6
*EP(3) 902*	*kay* (as) or *cg1973* (as)	34	64	25.1±1.8	25.3±12.2
*EP(3)614*	*cg12173*	51	63	27.2±1.3	25.5±17.4
*EP(2)506*	*HSPC300*	51	61	26.6±1.2	27.4±14.8
*EP(3)3617*	*miR-282*	36	61	27±1.4	32.7±17
*EP(2)323*	*cg8735*	35	61	26,7±1.1	24.7±11.3
*EP(3)662*	*slimb*	45	60	26.6±1.2	31.8±13.2
*EP(2)2345*	*dap* (as) or *cg10459* (as)	44	59	27±1	26±17
*EP(3)3084*	*kay*	29	59	23.9±1.2	28±14.3
*EP(2)2319*	*cg10082*	46	59	26.3±1.1	44±28.3
*EP(2)575*	*Rapgap1 (as) or cg13791* (as)	36	58	26.3±1.2	27±14.7
*EP(2)813*	*wech* (as) or *cg1621* (as)	24	58	27.2±1	31.2±13.2
*EP(3)1141*	*sda*	35	57	26.4±1.4	25.4±14.9
*EP(3)1110*	*cg9801*	27	56	26.7±1.9	26.7±9.3
*EP(2)2241*	*Dg*	23	56	26.4±0.7	30.3±10.8
*EP(3)661*	*cpo*	34	56	27±1.2	41.8±20.9
*EP(2)2469*	*kis* (as)	36	56	24.9±3.1	34.9±16.4
*EP(2)2254*	*akap200*	43	55	26.4±1.1	27.1±14.2
*EP(3)996*	*cg31184/cg33108*	36	53	26.5±1.5	29.3±14.3
*EP(2)2356*	*miR-310/311/312//313*	34	53	complex	
*EP(2)2098*	*cg30152*	35	51	26.6±1	29.5±16
*EP(2)2402*	*miR-8*	35	51	23.5±0.8	27.8±14.3
*EP(3)3094*	*lk6*	20	50	28.5±2.2	22.9±8.9
Controls					
*y w; tim-GAL4/+*		59	1.7	25.1	17
*cry^b^*		76	83	24.1±0.7	40.2±21

(as = antisense orientation).

In some lines, we occasionally observed one or two flies with complex behavior. Most of them displayed two components in their circadian behavior, one with a periodicity of 24 hr, and the other with 26.5 hr. This complex behavior was very rare, and probably occurred randomly. However, one line (*EP(2)2356/miR-310-311-312-313*) was strikingly different: about one quarter to a third of rhythmic flies showed a complex behavior when crossed to *tim-GAL4* (Table S2). The short component had a periodicity that varied between 18 and 22 hr, while the long component varied from 26 to 29 hr, or more rarely was around 24 hr. The other rhythmic flies from this line showed only one component, mostly 18–22 hr, with a few individuals around 24 hr or 26–29 hr (Table S2). This behavior is to our knowledge unique. Complex behavior has been observed in *cry^0^* flies, as well as in *cry^b^* and wild-type flies under specific light conditions, but the short period component had a period of approximately 23 hours [32]–[34].

### Function of the candidate genes

Virtually all *EP* insertions of the collection have been mapped to the genome (see Flybase, http://www.flybase.org). We verified the insertion location for eight lines, which were all identical to the insertion sites given in Flybase. For two genes (*lk6* and *morgue*), we also verified that they were indeed overexpressed as predicted (data not shown). We can therefore predict which genes are misexpressed in the selected lines (Table 1).

Among the candidate genes identified in our screen, we found seven genes that regulate gene expression (Table 2). Among them were three transcription factors. *elB* is known for its role in trachea and appendage development [35],[36]. *kay* is the *Drosophila* homologue of *fos* and is implicated in several signal transduction pathways. KAY is for example essential during embryonic development, for the differentiation of R3/R4 photoreceptors and in immune responses [37]–[42]. *kis* encodes a chromatin-remodeling protein of the Trithorax family [43],[44]. *cpo* was also identified in our screen (*EP(3)661*). A second *EP* line affecting *cpo* (*EP(3)3611*) was also detected initially, but its phenotype was weaker and thus not listed in Table 1. CPO is an RNA binding protein that regulates different aspects of *Drosophila* behavior (flight, phototaxism, negative geotactic behavior for example) [45].

**Table 2:** Candidate genes sorted by biological function.

Biological function	Genes	Molecular activity
Regulation of gene expression	*elB*	Transcription factor
	*kay*	Transcription factor
	*kis*	Transcription factor
	*cpo*	RNA binding protein
	*miR-282*	MicroRNA
	*miR-310-313*	MicroRNA
	*miR-8*	MicroRNA
Protein degradation	*morgue*	E2 ubiquitin conjuguase
	*slimb*	E3 ubiquitin ligase
	*calpB*	Calcium activated protease
	*sda*	Protease
Protein modification	*lk6*	Serine/threonine kinase
	*cg9801*	Serine/threonine phosphatase
Cytoskeleton regulation	*HSPC300*	
	*Dg*	
Protein localization	*Akap200*	PKA interacting protein, actin binding
Metabolism	*Gst-S1*	Glutathione-S-transferase
	*cg12173*	Aminoacid metabolism
	*cg10082*	Inositol metabolism
No putative function	*cg30152*	
	*cg8735*	

Only the genes that are unambiguously identified are mentioned in this table.

An interesting set of lines that resulted in LL rhythmicity affected microRNA genes (*EP(3)3041/miR-282*, *EP(3)714/miR-282*, *EP(3)3718/miR-282*, *EP(3)3617/miR-282*, *EP(2)2402/miR-8*, *EP(2)2356/miR-310-311-312-313*). Strikingly, among the six lines with the strongest phenotype were three lines (*EP(3)3041*, *EP(3)714* and *EP(3)3718*) that are predicted to overexpress *miR-282*. A fourth line (*EP(3)3617*) affects the same gene, and also showed a robust phenotype.

A second category of interesting candidate genes regulates protein stability. Two genes are implicated in proteasomal degradation (*morgue* and *slimb*). MORGUE has an E2 ubiquitin conjuguase domain [46], and SLIMB is an E3 ubiquitin ligase implicated in the control of PER levels [47]–[49]. In addition, overexpressing the E3 ubiquitin ligase complex subunit JET also results in LL rhythms (Figure 1A). Since these three proteins carry an F-box, we wondered whether the overexpression of any protein with an F-box or involved in proteasomal degradation could render flies resistant to LL. This was not the case: overexpressing the ubiquitin conjuguase UBCD1 or the F-box containing E3 ligase AGO did not result in LL rhythmicity (Figure S1). This demonstrates the specificity of the constant light phenotype to MORGUE, SLIMB and JETLAG. Two additional candidate genes encode proteases. CALPB is a calcium activated protease of the calpain family [50], whereas another protease, SDA, is known to be important for normal nervous system function: *sda* mutants are prone to seizures [51]. A kinase (*lk6*) involved in the control of growth through its action on the translation initiation factor 4E [52],[53] and a putative protein phosphatase (*cg9801*) were also isolated.

For several lines, the identity of the targeted gene was unclear. Two lines were located in front or within a genomic region with two overlapping genes. One of them (*EP(3)703*) was among the lines with the strongest behavior. The two overlapping genes encode a putative metalloprotease (*cg8176*) and a putative transcription factor of the jumanji family (*cg8165*).

Finally, for five *EP* lines (*EP(2)575*, *EP(3)902*, *EP(2)2345*, *EP(2)813* and *EP(2)2469*) the targeted genes are not up-regulated, but probably down-regulated, since the *P*-element is predicted to produce an antisense RNA. For *EP(2)575*, *EP(3)902*, *EP(2)2345* and *EP(2)813*, the identity of the gene which might be down-regulated is uncertain, because these *EP*-elements are located near the very 5′end of the *Rapgap1*, *kay*, *dap* and *wech* genes, respectively. They might thus affect the genes located 5′ of these three genes. *EP(2)2469* is inserted in the 12^th^ intron of the *kis* gene. It could also potentially overexpress *cg13693*, which is nested 10kb downstream of the *EP*-element, within *kis* 4^th^ intron. Evidence presented below will demonstrate that *kis* downregulation is sufficient to explain the LL phenotype obtained with *EP(2)2469*, although we cannot entirely exclude a contribution of *cg13693* overexpression to this phenotype.

### Phase responses to short light pulses are disrupted with a small subset of *EP* lines

**Figure 2:**
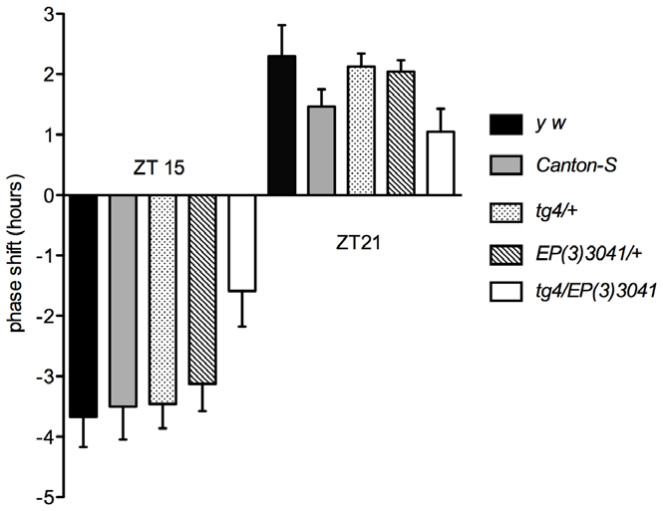
Flies overexpressing *miR-282* respond poorly to short light pulses. Phase responses to short light pulses in control flies (*y w, Canton-S, y w; tim-GAL4; +* [*tg4/+*] *and y w; +; EP(3)3041* [*EP(3)3041/+*]) and flies overexpressing the microRNA *miR-282* (*y w; tim-GAL4/+; EP(3)3041/+* [*tg4/EP(3)3041*]). Flies were entrained for 4 days to a 12hr∶12hr light∶dark cycle. During the fourth night, they were light-pulsed for 5 min at 1,000 lux at ZT15 and ZT21 and then left in constant darkness. Phase changes were calculated by comparing the phase of the evening peak in light-pulsed versus non-pulsed flies of the same genotype. Phase changes are plotted on the *y* axis; phase delays and advances are shown by negative and positive values, respectively. The histogram represents the average of three independent experiments (16 flies per experiment); the error bars represent standard deviations. *tim* variants: *y w* is homozygous for *s-tim*; *Canton-S* and *tg4/EP(3)3041* are homozygous for *ls-tim*; *tg4/+* and *EP(3)3041/+* are *ls-tim/s-tim* heterozygotes.

To determine how profoundly misexpression of the candidate genes disrupts the CRY input pathway, we tested the response to short light pulses, which is entirely CRY-dependent [16]. In wild-type flies, a 5-min light pulse induces a phase delay when administered at the beginning of the night and a phase advance when administered at the end of the night [54],[55]. All *EP* lines selected from the primary screen were challenged with 5-min light pulses at ZT15 and ZT21. Three *EP* lines responded poorly to light pulses at high intensity (1000 lux): *EP(2)2367/morgue*, as described previously [29], *EP(3)3041/miR-282* and *EP(3)714/miR-282* (Figure 2 and data not shown). For *EP(3)3041/miR-282* we observed a clear reduction of the response to light at ZT15 compared to all controls. At ZT21, we also observed a reduction, although the magnitude of the phase shift was not significantly different from that of one control (*Canton-S*). A similar phenotype was observed with *EP(3)714/miR-282* (data not shown). The other *EP* lines responded normally to short light pulses. Previous studies have shown that among CRY dependent photoresponses, the behavioral responses to constant light are far more sensitive to partial disruption of the CRY input pathway [18],[23]. Thus, it appears that *morgue* and *miR-282* overexpression have the most profound effect on CRY signaling, while overexpression of the other candidate genes affect the CRY input pathway more moderately. As mentioned above, most of the *EP* lines show a long period phenotype and a weak power, which demonstrate that their circadian clock is not insensitive to light. Strikingly, the three lines that disrupt phase responses to short light pulses are among those with the strongest phenotype in LL.

### KIS is essential for normal circadian photoresponses to constant light

To identify positive regulators of CRY signaling among our candidate genes, we screened loss-of-function mutants under constant light (200 lux). For 5 candidate genes, we tested severe loss-of-function alleles that were homozygous viable and healthy (*lk6*, *morgue*, *akap200*, *GstS1* and *sda*, see Materials and Methods). They were all completely arrhythmic in LL (data not shown). For most candidate genes however, severe loss-of-function mutations were either lethal or not available. We therefore decided to use RNA interference, targeted specifically to circadian tissues, which should in most cases downregulate candidate gene expression and avoid lethality. We crossed *tim-GAL4* flies to transgenic flies from the VDRC and NIG-Fly RNAi collections, which carry transgenes encoding gene-specific double-stranded RNAs (dsRNAs). Lethality was only observed with three candidates: *kay*, *cpo* and with one of the RNAi line targeting *elB*. Most viable *tim-GAL4*/dsRNA flies were arrhythmic in LL. However, two lines targeting the chromatin remodeling gene *kismet* (*NIG-Fly3696R-1* and *VDRC46685*) were 50–60% rhythmic, with a power of about 35 (Table 3). These results are strikingly similar to those obtained with *EP(2)2469/kismet*, which as mentioned above is inserted in an antisense orientation within *kis'* 12^th^ intron, and is thus predicted to generate a *kis* antisense RNA. The only noticeable difference in behavior between the two RNAi lines was that *NIG-Fly3696R-1* shows a period close to 24 hr, whereas *VDRC46685* and the *EP* line have a ca. 26 hr period rhythm. This could indicate that the NIG-Fly line is more efficient at repressing KIS expression. In DD, both *kis* RNAi lines were rhythmic with a period close to that of control flies (Table S3).

**Table 3:** Behavior of flies expressing *kismet* dsRNAs in constant light.

Genotype	*tim* alleles	% of rhythmic flies	period average ±SD	Power average ±SD	n
*y w*	*s-tim/s-tim*	0.00%			106
*y w; NIG-Fly3696R-1/+*	*s-tim/s-tim*	5.45%	22.12±1.48	16.52±2.42	110
*y w; tim-GAL4/+*	*ls-tim/s-tim*	12.73%	25.31±5.11	20.28±6.39	110
*y w; tim-GAL4/+; NIG-Fly 3696R-1/+*	*ls-tim/s-tim*	59.57%	24.16±1.66	35.08±18.95	94
*y w; VDRC46685/+*	*ls-tim/s-tim*	6.25%	21.93±1.29	17.57±7.15	48
*y w; VDRC46685/+*	*ls-tim/ls-tim*	6.25%	21.0	18.25	16
*y w; tim-GAL4/+*	*ls-tim/ls-tim*	17.39%	21.93±1.29	17.57±7.15	23
*y w; tim-GAL4/VDRC46685*	*ls-tim/ls-tim*	65.96%	26.18±1.24	25.43±6.29	47
*y w; tim-GAL4/VDRC46685*	*ls-tim/s-tim*	50.00%	24.96±1.22	24.28±13.17	16
*w^1118^; tim-GAL4, UAS-dcr2/+*	*ls-tim/ls-tim*	9.38%	26.47±0.40	26.30±8.61	32
*w^1118^; tim-GAL4, UAS-dcr2/VDRC46685*	*ls-tim/ls-tim*	82.76%	25.14±0.97	51.02±29.27	29
*w^1118^; tim-GAL4, UAS-dcr2/NIG-Fly3696R-1*	*ls-tim/s-tim*	62.50%	24.25±0.93	35.35±17.90	32
*y w; cry^b^/cry^b^*	*ls-tim/ls-tim*	76.58%	24±0.53	62.04±26.92	111

**Figure 3:**
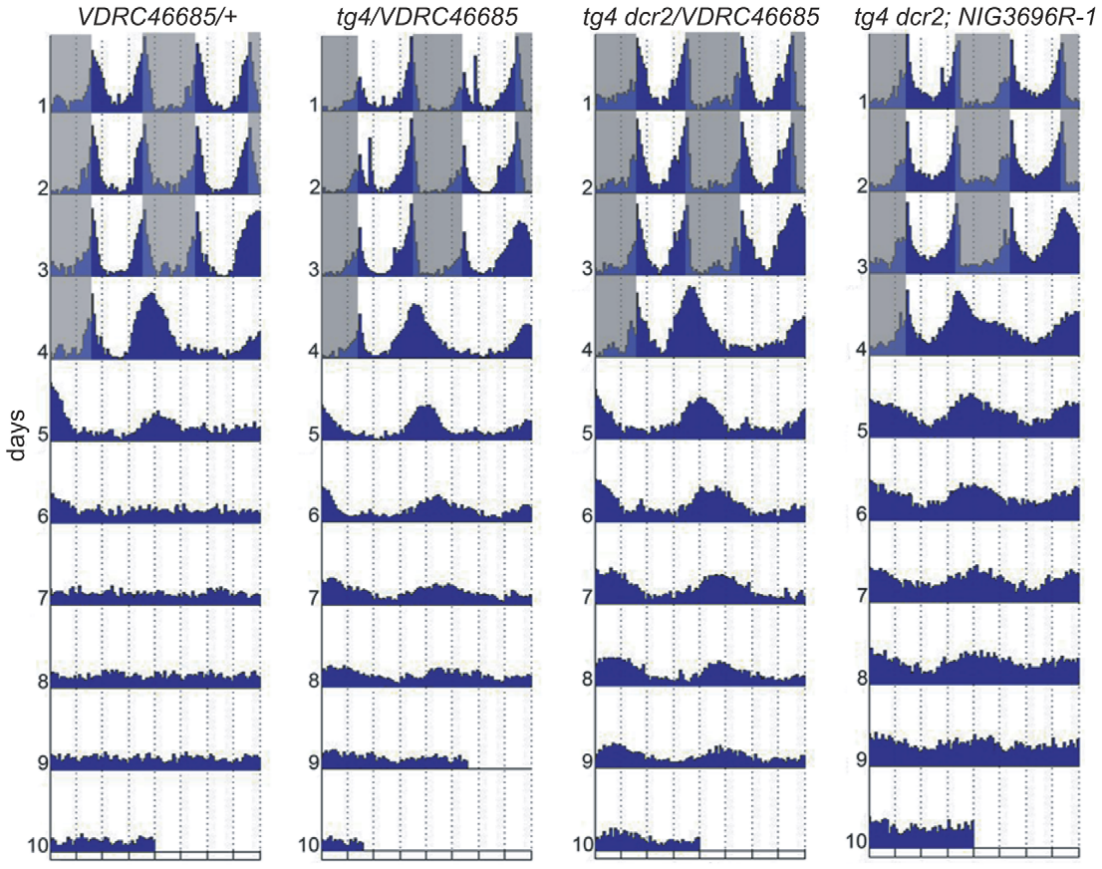
Flies expressing double-stranded RNAs directed against *kismet* (*kis*) in circadian neurons are rhythmic in LL. The actograms show the average locomotor behavior of control flies (*w^1118^*; *VDRC46685/+* [*VDRC46685/+*]) and flies expressing *kis* dsRNAs under the control of *tim-GAL4* without (*y w; tim-GAL4/VDRC46685* [*tg4/VDRC46685*]) or with *dicer2* (*w^1118^; tim-GAL4 UAS-dcr2/VDRC46685* [*tg4 dcr2/VDRC46685*] and *w^1118^; tim-GAL4 UAS-dcr2; NIG-Fly3696R-1* [*tg4 dcr2; NIG3696R-1*]). Flies were entrained for 3 days in LD and then released in LL. Note that rhythms are more robust when *dicer2* is coexpressed with the *VDRC46685 kis* dsRNAs. All genotypes are homozygous for *ls-tim*, except *tg4 dcr2; NIG3696R-1* (*ls-tim/s-tim*). n = 15–16 flies/genotype.

Since *kis* RNAi flies are clearly less robustly rhythmic than *cry^b^* flies, we co-expressed *dicer2* along with the *kis* dsRNAs, to increase the RNAi effects [56]. This approach proved successful, as flies expressing the VDRC dsRNAs directed against *kis* were almost as robustly rhythmic as *cry^b^* flies (Table 3, Figure 3). The period was also similar to that observed in DD (Table S3), further indicating that these flies are virtually insensitive to constant illumination and that KIS is essential for normal circadian photoresponses. As the period length and amplitude of DD rhythms are not affected by KIS downregulation (Table S3), KIS specifically regulates circadian photoresponses.

**Figure 4:**
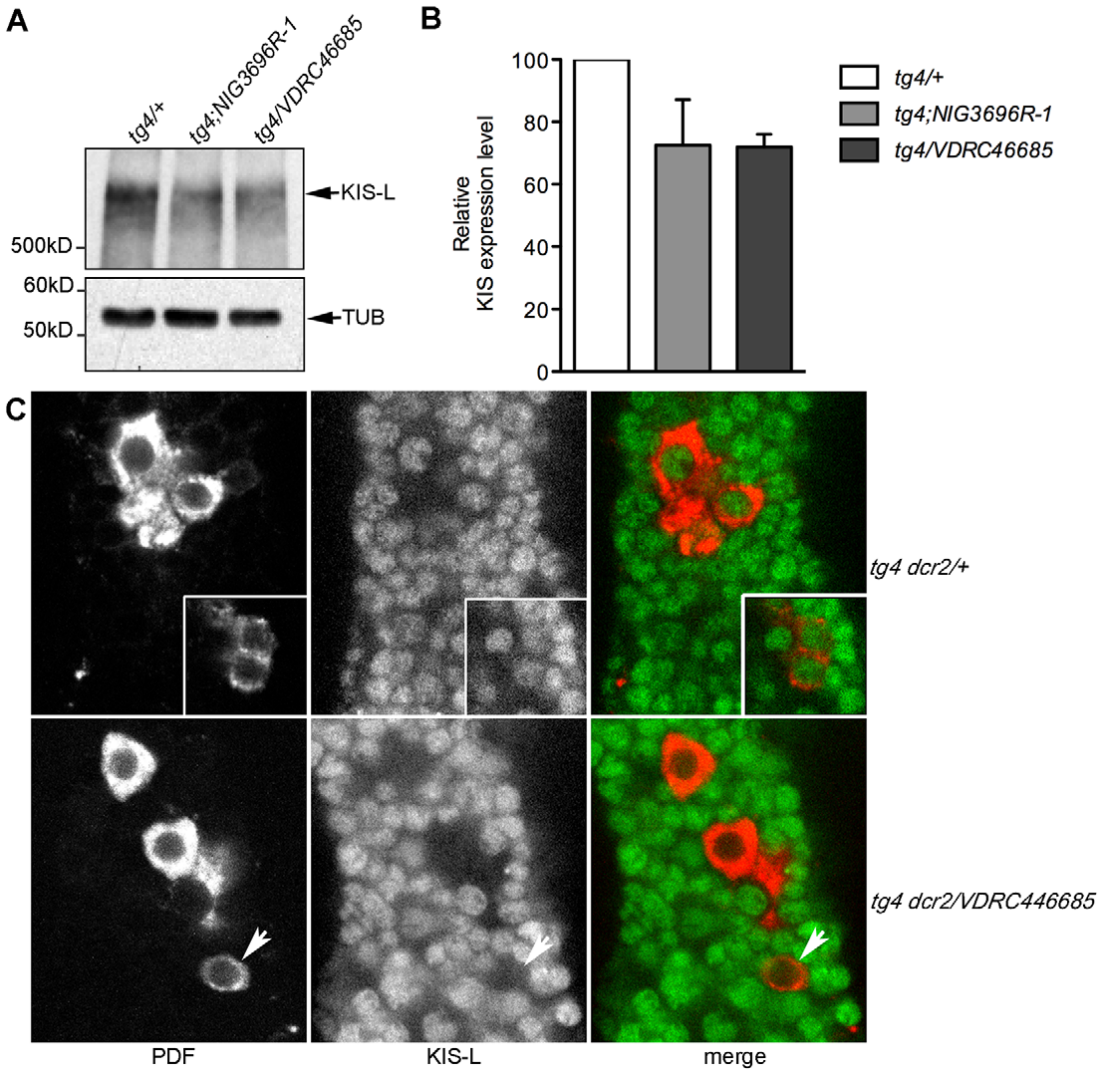
*kismet* is downregulated in flies expressing *kis* dsRNAs. (A) Representative Western Blot measuring KIS-L protein levels in whole head extracts of control flies (*tim-GAL4/+* [*tg4/+*]) and flies expressing two different dsRNAs directed against *kis* (*NIG-Fly3696R-1* [*NIG3696R-1*] and *VDRC46685*) under the control of *tim-GAL4* (*tg4*). (B) Quantification of three independent Western Blots. KIS-L levels were normalized with the Tubulin loading control. Error bars correspond to standard deviations. KIS-L is reduced by about 30% in whole head extracts of flies expressing *kis* dsRNAs, compared to control. (C) Images obtained by confocal microscopy showing the s-LN_v_s (shown in the insert on the upper panels and indicated by an arrowhead on the lower panels) and l-LN_v_s of *tim-GAL4 UAS-dcr2/+* controls (*tg4 dcr2/+*; upper panels) and of *tim-GAL4 UAS-dcr2/VDRC46685* (*tg4 dcr2/VDRC46685*) flies (lower panels). The brains were stained with an anti-PDF antiserum (red) and an anti-KIS-L antibody (green). KIS-L is almost undetectable in the nuclei of s- and l-LN_v_s of flies expressing the *kis* dsRNAs, while it is strongly expressed in non-circadian neighbor neurons (lowers panels), and in the s- and l-LN_v_s of control flies (upper panels). See Figure S2 for quantifications.

A major potential caveat with expression of dsRNAs is off-target effects: dsRNAs sometimes target other genes than the one they were designed to downregulate. With *kis*, we have observed a similar phenotype with two independent RNAi transgenes that target two different regions of the *kis* RNA. In addition, *EP(2)2469/kismet* targets a third region of the *kis* gene specific to the KIS-L isoform, which contains most domains necessary for KIS function in chromatin remodeling [43]. Thus, the risks of off-target effects are virtually non-existent. In addition, no potential off-targets are predicted for *VDRC46685* (http://stockcenter.vdrc.at/control/main). Nonetheless, we verified that the RNAi lines we used indeed downregulate KIS expression. We first measured KIS expression in whole heads by Western Blot using the *tim-GAL4* driver to induce *kis* RNAi, and found KIS levels to be reduced by 30% with both RNAi lines (Figure 4A and 4B). Since the dsRNAs were expressed with *tim-GAL4*, this highly reproducible reduction in KIS expression should reflect the downregulation occurring specifically in circadian tissues, in particular the eyes which are by far the largest contributors of circadian tissues in whole heads [57]. KIS should not be down-regulated in non-circadian tissues.

There are ∼150 circadian neurons in the *Drosophila* brains that are organized in 5 clusters in each hemisphere: the large and the small ventral lateral neurons (l-LN_v_s and s-LN_v_s), which express the neuropeptide PDF, the dorsal lateral neurons (LN_d_s) and three groups of dorsal neurons (DN1s, 2s and 3s). We verified that KIS-L expression was indeed specifically down-regulated in circadian neurons by staining brains from flies expressing *kis* dsRNAs in clock tissues and control flies with an antibody directed against KIS-L [43] along with an anti-PDF antibody. KIS-L was strongly expressed in the LN_v_s of wild-type flies, as well as in non-circadian neurons. KIS-L levels were unaffected in non-circadian neurons of flies expressing the dsRNAs with *tim-GAL4*, but KIS-L expression was severely downregulated in the LN_v_s (Figure 4C, Figure S2A).

In summary, our results identify KIS as a crucial regulator of CRY-dependent circadian photoresponses. This validates our screen strategy.

### KISMET functions in circadian neurons regulating circadian light responses

**Figure 5:**
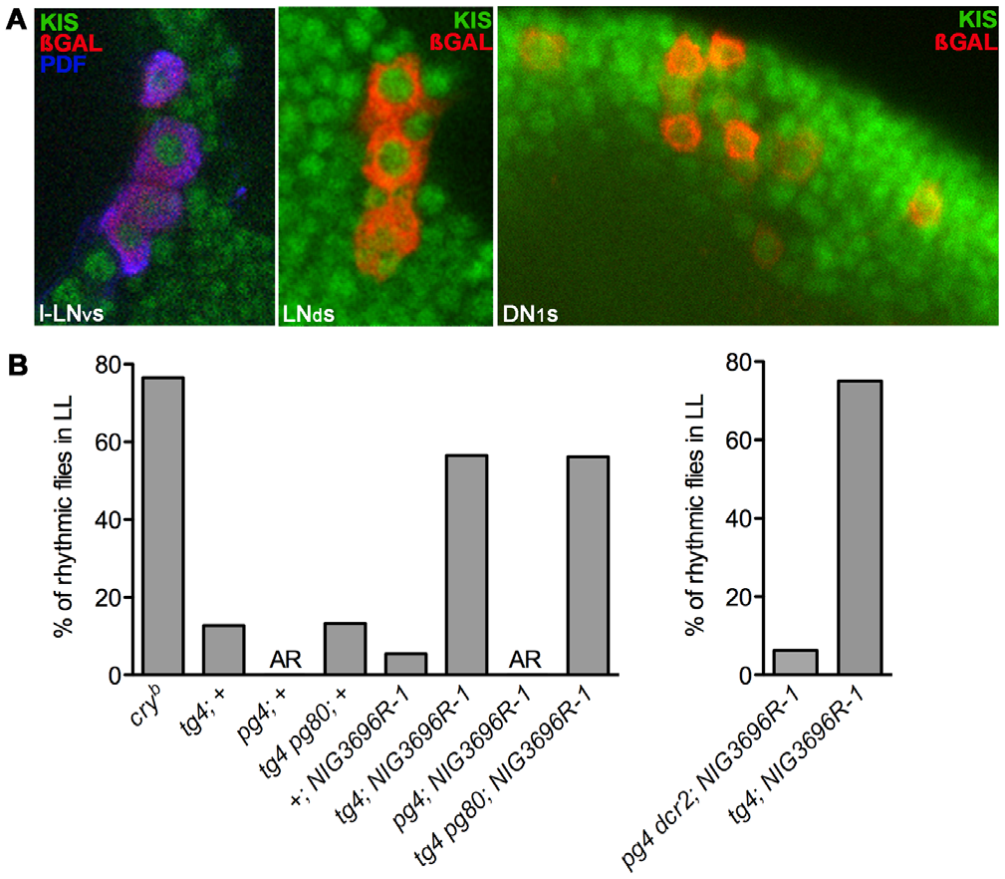
KIS is expressed and functions in PDF negative circadian neurons to control behavioral responses to constant light. (A) Whole brains of flies expressing *lacZ* in all circadian neurons (line R32 [58]) were stained with anti-ßGAL (red), anti-PDF (blue) and anti-KIS-L (green) antibodies. The purple color indicates the colocalization of ßGAL and PDF. KIS is detected in the nuclei of the LN_v_s, LN_d_s and DN1s. (B) *kis* was downregulated in different groups of circadian neurons using a combination of tissue-specific *GAL4* and *GAL80* drivers. Left panel: The expression of *kis* dsRNAs (*NIG-Fly3696R-1* [*NIG3696R-1*] carrying *s-tim*) was driven by either *tim-GAL4* (*tg4;* carries *ls-tim*) to down-regulate *kis* in all circadian neurons, *pdf-GAL4* (*pg4;* carries *s-tim*) to restrict *kis* RNA interference to the LN_v_s, or with *tim-GAL4* combined with *pdf-GAL80* (*tg4 pg80*; *ls-tim*) to downregulate KIS expression in PDF negative circadian neurons only. Right panel: Since *pdf-GAL4* is inserted on a second chromosome containing a *s-tim* allele while *tim-GAL4* is associated with a *ls-tim* allele, we also drove KIS dsRNAs expression with a *pdf-GAL4 UAS-dcr2* (*pg4 dcr2*) recombinant 2^nd^ chromosome bearing a *ls-tim* allele. This ensured that the absence of LL rhythms with *pdf-GAL4* is not caused by the more sensitive *s-tim* variant. The histograms show the percentage of rhythmic flies for each genotype in LL (n = 14–16). AR: complete arrhythmicity. Left and right histograms are independent experiments; this explains the small variation in rhythmicity with *tim-GAL4*.

Since KIS regulates constant light responses, it should be expressed in circadian neurons known to control these responses. To identify easily circadian neurons, we stained brains expressing a *lacZ* reporter gene in all clock neurons (line R32, [58]) with antibodies directed against ß-GAL and KIS-L, because our results indicate that this isoform regulates light responses (see above). We found KIS-L to be expressed in l- and s-LN_v_s, in the LN_d_s, and in DN1s and 3s (Figure 5A). Most of these neurons have been implicated in circadian behavioral photoresponses [26], [29], [33], [59]–[65]. In particular, the LN_d_s and DN1s have been shown to be able to generate LL rhythms when the CRY input pathway is inhibited in these cells. That KIS is expressed in these cells, and that KIS downregulation results in LL rhythms clearly support the idea that KIS is a crucial regulator of the CRY input pathway.

We were therefore curious to verify that behavioral rhythms observed under constant illumination in *kis* RNAi flies were due to KIS downregulation in dorsally located circadian neurons, and not in the PDF positive LN_v_s. We thus used the *pdf-GAL4* driver [66] to restrict *kis* RNAi expression to the LN_v_s, and a combination of *tim-GAL4* and *pdf-GAL80* to express *kis* dsRNAs in dorsal neurons only [67]. *kismet* down-regulation in the LN_v_s alone did not affect behavior in LL: all flies were arrhythmic. On the other hand, flies expressing *kis* dsRNAs in PDF negative circadian neurons were rhythmic in LL (Figure 5B). The absence of LL rhythms when driving *kis* dsRNAs only in the LN_v_s is not due to inefficient KIS downregulation: *pdf-GAL4* and *tim-GAL4* are equally efficient at downregulating KIS expression (Figure S2A). Together, these results demonstrate that KIS activity is required in PDF negative light-sensitive circadian neurons for normal CRY-dependent circadian photoresponses.

### *kis* genetically interacts with *cry* and regulates TIM degradation

**Figure 6:**
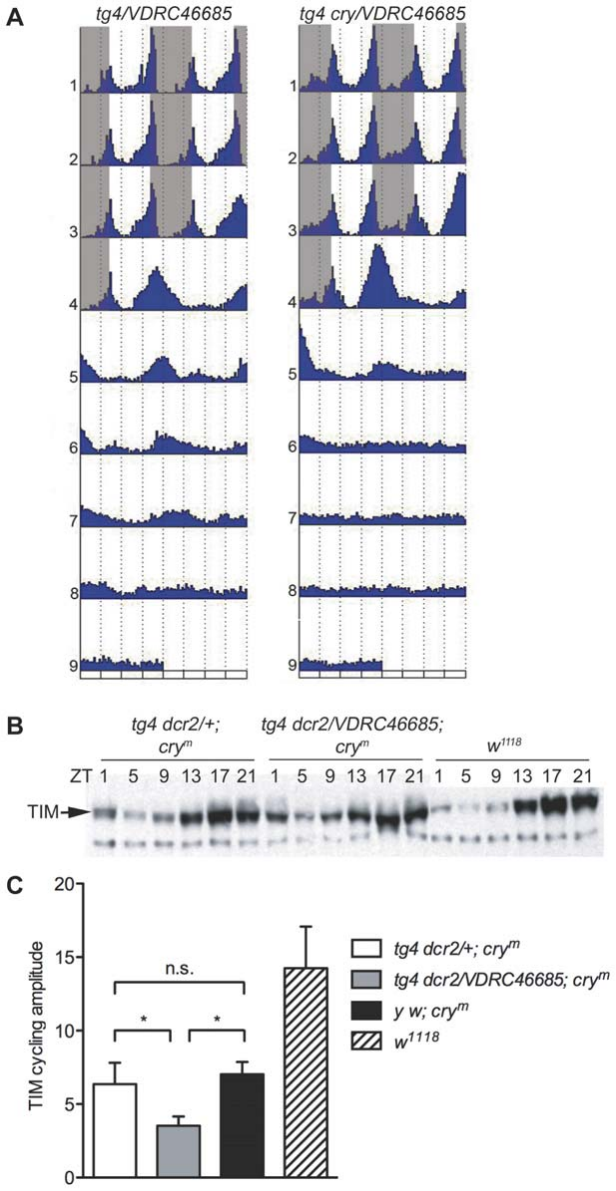
*kismet* and *cry* genetically interact. (A) Increasing CRY signaling in flies expressing *kis* dsRNAs rescues the LL phenotype. Actograms show the locomotor activity of *tim-GAL4/VDRC46685* (*tg4/VDRC46685;* homozygous for *ls-tim*) and *tim-GAL4 UAS-myccry/VDRC46685* flies (*tg4 cry/VDRC46685; s-tim/ls-tim* heterozygous). The presence of one *s-tim* allele in flies overexpressing CRY potentially contributes to the rescue, because the S-TIM protein is more sensitive to CRY signaling [87]. This contribution is small however. Indeed, 50% of *tim-GAL4/VDRC46685* flies carrying one *s-tim* allele are rhythmic in constant light (see Table 3). In addition, CRY overexpression also rescues arrhythmicity in a *ls-tim* homozygous background, although a slight increase in residual rhythms was detected during the first 3 days of LL (Figure S3). n = 15–16 flies/genotype. (B) Western blot showing TIM oscillations in whole head extracts of control (*w^1118^*), *timGAL4 UAS-dcr2*/+; *cry^m^/cry^m^* (*tg4 dcr2*/+; *cry^m^*) and *tim-GAL4 UAS-dcr2/VDRC46685; cry^m^/cry^m^* (*tg4 dcr2/VDRC46685; cry^m^*) flies entrained to an LD cycle. All genotypes are homozygous for *ls-tim*. (C) The amplitude of TIM protein rhythms is reduced in *cry^m^* flies expressing *kis* dsRNAs. TIM protein levels were quantified by Western Blot at ZT17 (peak) and ZT5 (trough) and normalized with the Tubulin loading control. The histogram represents the ratio between the two values. Each bar represents the average of 4 independent experiments per genotypes, expect *y w; cry^m^* (n = 3). Error bars represent standard deviations (*p<0.05; t-test).

CRY targets TIM to proteasomal degradation [16],[20],[21]. The prediction would therefore be that if KIS regulates the CRY input pathway, light-dependent TIM degradation would be inhibited by KIS downregulation. Unexpectedly, we did not observe any defect in TIM light-dependent protein cycling in head extracts of flies expressing *kis* dsRNAs (data not shown). This is probably because RNA interference does not completely abolish KIS expression in the eyes, which are the main contributor of TIM protein in head extracts. Since CRY levels appear to vary significantly in different circadian tissues [68]–[70], we reasoned that CRY (or its signals to the pacemaker) might be limiting in the PDF negative neurons generating LL rhythms (LN_d_s, DN1s), but not in the eyes. This would explain why LL behavior is particularly sensitive to KIS levels. We therefore overexpressed CRY in all clock neurons of flies expressing *kis* dsRNAs. As we anticipated, this genetic manipulation rescued the behavioral phenotype resulting from KIS knockdown: the double-manipulated flies behaved like wild-type flies as they became arrhythmic after their release in LL (Figure 6A and Figure S3). Thus, when CRY is not limiting, circadian photoresponses are much less sensitive to KIS levels.

We therefore decided to express *kis* dsRNAs in *cry^m^* mutant flies [18]. The *cry^m^* allele encodes a truncated form of CRY lacking the last 19 amino acids of its C-terminal domain. This truncated CRY is very unstable but remains functional. In *cry^m^* flies, TIM levels oscillate in LD but the amplitude of this molecular rhythm is significantly reduced relative to wild-type flies, because CRY levels are limiting [18] (Figure 6B and 6C). Interestingly, when we expressed *kis* dsRNAs in *cry^m^* flies, TIM cycling amplitude was further reduced to about half of what it normally is in *cry^m^* flies. This effect was highly reproducible and statistically significant (p<0.05, t test). Notably, TIM levels remained higher during the day, even though during the night they were reduced. Note that TIM cycling is not sensitive to eye color: no difference was observed in amplitude between the white-eyed *cry^m^* flies (*y w; cry^m^*) and deep orange-eyed *cry^m^* flies (*y w; tim-GAL4 UAS-dcr2/+; cry^m^*; Figure 6B and 6C). Thus, we are confident that the addition of the *kis* dsRNA transgene (in *y w; tim-GAL4 UAS-dcr2/VDRC46685; cry^m^)*, which only very slightly darkens eye color compared to control flies (*y w; tim-GAL4 UAS-dcr2/+; cry^m^*), does not contribute to the reduction in TIM cycling amplitude. Moreover, in *cry^+^* flies, CRY and TIM levels are unaffected by this slightly darker eye color (data not shown).

In summary our results demonstrate that *kis* interacts genetically with *cry* and regulates circadian photoresponses not only in brain pacemaker neurons, but also in peripheral oscillators such as the eyes.

## Discussion

Our constant light screen has identified a novel important regulator of circadian photoresponses: KISMET (KIS). Our results show that KIS is essential for a well-characterized CRY-dependent circadian photoresponse: constant light (LL) induced behavioral arrhythmicity. This arrhythmic behavior is caused by the persistent activation of CRY by blue-light photons. CRY thus binds constantly TIM and tags it for JET-mediated proteasomal degradation. This leaves PER unprotected from being itself targeted to proteasomal degradation, and leads to the disruption of the molecular pacemaker in neurons controlling circadian behavior. Consistent with previous studies in which the CRY input pathway was partially disrupted [18],[23], we observed that among CRY-dependent light responses, LL arrhythmicity is much more sensitive to reduction in KIS expression. Our results further suggest that CRY levels are limiting in circadian neurons that can generate LL behavior. It is becoming clear that CRY level varies significantly between circadian neurons [69],[70], and it is therefore likely that those with lower CRY levels are more prone to become rhythmic in LL when the CRY input pathway is partially disrupted (this study and [26],[29]). We presume that this reflects the very fine photosensitive tuning needed for CRY photoresponses. The circadian input pathway has to be able to respond to photoperiod length and to progressive light intensity changes at dawn and dusk, but at the same time should not respond inappropriately to moonlight.

KIS role as a regulator of the CRY input pathway is not limited to circadian neurons. It also influences circadian photoresponses in peripheral circadian tissues such as the eyes, since we observed a significant reduction in diurnal CRY-dependent TIM protein oscillations in *cry^m^* flies expressing *kis* dsRNAs. Whether KIS is actually essential for light responses in every circadian tissue is not yet clear. Indeed, we were limited to use RNA interference - which usually does not completely abolish gene expression - to study KIS function in adult flies, because *kis* null mutants are lethal. Incomplete KIS knockdown might explain why we could not detect any defects in the phase response to short light pulses (data not shown). The neurons controlling this circadian photoresponse are at least partially distinct from those controlling LL behavioral responses [59]; they may have retained sufficient residual KIS or having high CRY levels. Alternatively, KIS might not be essential in these circadian neurons.

KIS is a chromatin-remodeling enzyme that was initially discovered in a screen for extragenic suppressor of Polycomb mutations [71]. It was thus categorized as a Trithorax protein, a group of transcriptional activators of homeotic genes that counteracts Polycomb negative regulators. Recent evidence obtained with larval salivary glands has suggested that KIS might be a general regulator of transcription [43]. Indeed, KIS is associated with most, but not all, transcriptionally active sites of larval salivary gland polytenic chromosomes. In *kis* mutants, RNA Polymerase II is associated with these sites, but remains hypophosphorylated, which indicates that it is unable to initiate mRNA elongation. In addition, elongation factors are not recruited at transcriptionally active sites. It was therefore proposed that KIS is necessary for the recruitment of these elongation factors and for reorganizing chromatin downstream of the transcriptional start site [43],[72]. However, while we observed strong effects on circadian light responses, we did not detect any significant effects on the period of the circadian oscillator. Our results therefore indicate that KIS is specifically involved in the control of circadian light input genes in neurons controlling circadian light responses. Since we have used RNA interference to disrupt KIS expression, we cannot entirely exclude that residual KIS expression is sufficient for maintaining normal transcription of pacemaker and non-circadian genes. We also cannot exclude the possibility that other chromatin remodeling enzymes could substitute for KIS in the control of these genes. There is however very clear experimental evidence that supports the idea that KIS regulates the expression of specific genes. *kismet* loss-of-function results in specific segmentation defects and homeotic transformation during development [44]. Moreover, a recent study demonstrates that KIS plays a central and specific role in the regulation of *atonal*, a pro-neural gene, in the fly retina [73]. We therefore strongly favor the hypothesis that KIS is dedicated to the control of circadian light input genes in *Drosophila* circadian clock neurons. We do not know yet the identity of these genes. We have measured by Real-Time quantitative PCR the expression of the known components of the CRY input pathway (*cry*, *sgg*, *jet*, *tim*, *csn4*, *csn5*) but did not detect any significant change in their mRNA levels when KIS is knocked-down (data not shown). This indicates that important elements of the CRY input pathway remain to be identified. These proteins either function downstream of CRY or regulate CRY activity, but they apparently do not affect CRY abundance. Indeed, we could not detect any changes in CRY levels by Western Blots or brain immunostainings in flies expressing *kis* dsRNAs (data not shown).

The demonstration that KIS is essential for circadian light responses validates our screen for circadian light input genes, which has identified over 20 additional genes that might regulate circadian light responses. As most of these genes were overexpressed in the screen, a significant fraction of them might be negative regulators of the CRY input pathway. It is thus entirely possible that a reduction in their expression levels or a complete loss-of-function would result in an increase in light sensitivity, rather than a loss of CRY responses. Our loss-of-function subscreen was aimed at genes essential for the CRY input pathway and would not have detected genes that increase light sensitivity. It is therefore not surprising that we confirmed a gene (*kis*) that was predicted to be downregulated in our initial screen as essential for circadian photoresponses.

Future studies will determine whether other candidate genes are negative regulators of CRY signaling. They will also be aimed at determining whether some of the candidate genes might be part of the circadian pacemaker, rather than regulators of CRY signaling. This is entirely possible, since overexpression of circadian pacemaker genes such as PER or TIM results in LL rhythms [26],[29]. Actually, we identified one other pacemaker gene in our screen: *slimb* [48],[49]. The isolation of this gene is unexpected however. *slimb* overexpression would be predicted to reduce PER levels, since it promotes PER ubiquitination and proteasomal degradation. A possibility is that *slimb* overexpression is toxic to the PER degradation pathway, and thus results in an increase, rather than a decrease, in PER levels. This idea is supported by the fact that strong *slimb* overexpression results in the same circadian phenotype as *slimb* loss-of-function mutations: arrhythmic behavior in DD [48],[49]. Moreover, we observed that overexpressing *jet* - which is involved in proteasome-dependent protein turnover, like *slimb* [23] - disrupts circadian photoresponses in LL. This could also be explained by a dominant-negative effect of *jet* overexpression on TIM proteasomal degradation. However, recent results demonstrating that JET also promotes CRY proteasomal degradation [24] point at another potential explanation: CRY levels might be reduced when *jet* is overexpressed. In any case, the negative effect of *jet* overexpression on circadian light responses might explain why using the GAL4/UAS system to try to correct the photoreceptive defects of *jet* mutants resulted only in a partial rescue [23].

In addition to *slimb*, several other genes have been connected to circadian rhythms: *lk6*, *akap200*, *calpB* and *morgue*. The mRNAs of the last three genes were shown to cycle in the fly head in a DNA microarray study [74], while *lk6* was shown to oscillate in fly bodies [75]. RNase protection and Northern Blot assays revealed that *lk6* expression also undergoes circadian oscillations in heads, with a cycling phase and amplitude of oscillation similar to those of *cry* (Figure S4). Thus *lk6* and *cry* may be co-regulated. It might however be the presence of microRNAs in our screen that is most intriguing. MicroRNAs are known to play an important regulatory role in a variety of biological processes, which include development and neuronal function. In mouse, two miRNAs, *miR-219* and *miR-132*, have recently been shown to be under circadian regulation in the suprachiasmatic nucleus, and *miR-132* may be important in the regulation of photic responses [76]. In *Drosophila*, a role for miRNAs in the control of circadian rhythms has recently been demonstrated. In particular, *bantam* was shown to regulate CLK expression and thus to affect the amplitude of circadian rhythms [77]. In addition, a few miRNAs have been shown to be under circadian regulation [78], although their importance for circadian rhythms is currently unclear. None of the miRNAs we isolated are described to cycle in fly heads. However, two of them are expressed in circadian tissues: *miR-282* and *miR-8* [77]. This strongly supports the idea that these miRNAs are important for the regulation of circadian rhythms. *miR-282* appears particularly likely to be an important regulator of circadian photoresponses, since its overexpression affects profoundly both the behavior of the flies in LL and their response to short light pulse. Moreover, a predicted target of *miR-282* is *jetlag* (TargetScanFly, release4.2; Yong and Emery, unpublished observations), which is crucial for CRY signaling and TIM degradation. We are currently determining whether *miR-282* is indeed a regulator of the CRY input pathway.

In summary, our work has identified new candidate circadian genes. They might control or modulate circadian light responses and photosensitivity, or they might regulate circadian pacemaker function. Importantly, we have assigned a function to the chromatin-remodeling factor KISMET in adult flies: KIS control the photosensitivity of the circadian clock. The function of most chromatin-remodeling proteins is well documented during *Drosophila* development, but their function in the adult fly is not well studied, because null mutants for these genes are frequently lethal. The adult function of KIS was completely unknown, although we show here its expression in both circadian and non-circadian fly brain neurons. CHD7 is a human KIS homolog associated with CHARGE syndrome, a genetic disorder characterized by developmental retardation and complex abnormalities affecting several organs, including the brain and sensory systems [79],[80]. The partial loss of CRY signaling should be a powerful tool for a genetic screen aimed at finding KIS-interacting genes that contribute to transcriptional regulation. This might in turn reveal how CHD7 functions, and help illuminate the causes of CHARGE syndrome.

## Materials and Methods

### *Drosophila* stocks

The EP line collection was previously described [28]. The following strains were used: *y w*, *Canton-S*, *y w; tim-GAL4/CyO* [17], *y w; pdf-GAL4* [66]; *y w; cry^b^ ss* [16]; *y w; cry^m^* [18]; *y w ; tim-GAL4 pdf-GAL80/CyO; pdf-Gal80/TM6B* [29]; *y w; UAS-cry #12* [17]. The second chromosomes bearing *tim-GAL4* and *pdf-GAL4* insertions were independently and meiotically recombined with a chromosome containing the *UAS-dcr2* insertion [56]. The presence of the two transgenes on the same chromosome was verified by PCR. To localize the circadian neurons in *Drosophila* brain, the previously described enhancer trap line R32 was used [58]. We also used the following mutant strains: *sda^iso7.8^* [*51*], *Akap200^Δ7^* [*81*] *, lk6^1^ and lk6^2^* [*52*] *, Df(3R)exel9019, GstS1^M38-3^* and *GstS1^M29-13^* (Benes et al., unpublished GstS1 null mutants), *morgue^Δ457^* (Schreader and Nambu, unpublished deletion strain).

To induce RNAi of the candidate genes, the following lines were used from the NIG-Fly stock and the VDRC. NIG-Fly lines: 30152R-1 (*cg30152*), 30152R-2 (*cg30152*), 10459R-1 (*cg10459*), 3412R-1 (*slimb*), 3412R-2 (*slimb*), 15437R-1 (*morgue*), 15437R-2 (*morgue*), 15507R-2 (*kay*), 15507R-4 (*kay*). VDRC lines: 4024 (*cg8735*), 4025 (*cg8735*), 5647 (*akap200*), 11763 (*elB*), 42813 (*elB*), 14385 (*cpo*), 14691 (*cg1621*), 14692 (*cg1621*), 22144 (*sda*), 22145 (*sda*), 23037 (*calpB*), 46241 (*calpB*), 25033 (*cg31123*), 25034 (*cg31123*), 31674 (*cg1273*), 31676 (*cg1273*), 32885 (*lk6*), 38326 (*cg10082*), 38327 (*cg10082*), 38848 (*cg30152*), 41451 (*cg10459*), 41623 (*wech*), 35794 (*HSPC300*). *kismet* RNAi was induced using either the line 3696R-1 (NIG-Fly) or 46685 (VDRC). *UAS-ubcd1* was described in [82], and AGO overexpression was obtained with *EP(3)1135* [49].

Two alleles of *tim* can be found in lab stocks: *ls-tim* and *s-tim* [22]. *s-tim* is more sensitive to light than *ls-tim*. Our *y w* stock carries the *s-tim* allele, while *w^1118^* and *Canton-S* carry the less sensitive *ls-tim*. The 2^nd^ chromosome with the *tim-GAL4* insertion carries *ls-tim*, as well as the 2^nd^ chromosomes of *UAS-cry (line #12)*, *UAS-dcr2*, *EP(3)3041*, *EP(3)714*, *VDRC46685*, *y w*; *cry^b^*, *y w*; *cry^m^* stocks and *y w; pdf-GAL4 UAS-dcr2 (rec4)/CyO*. *NIG-Fly3696R-1* and the *tim-GAL4 UAS-myccry* recombinant chromosome carries *s-tim*. We also generated a *VDRC46685* stock with a *s-tim* allele, and a recombinant *tim-GAL4 VDRC46685* chromosome carrying the *ls-tim* allele. All these lines are wild-type for *jet*. Appropriate controls were included in all tables and figures to verify that the decrease in light sensitivity observed with *kis* downregulation or with *miR-282* overexpression was not due to *tim* variants.

### Behavioral analysis

#### Locomotor activity

1–5 day old single males were monitored for locomotion activity in Trikinetics Activity Monitors (Waltham, MA) for 3 full days under 12hr∶12hr light∶dark cycle followed by 6 full days of either constant light or constant darkness at 25°C in I-36LL Percival incubators (Percival Scientific, Perry IA). For the *EP* line screen, the light intensity was about 1000 lux, all other experiments were done at about 200 lux. Data were analyzed using FaasX software [61]. Rhythmic flies were defined by χ^2^ periodogram analysis with the following criteria: power ≥10 and width ≥2 [83]. Actograms were generated using a signal processing toolbox for Matlab (Math Works, Natick, MA) [84].

#### *EP* line screen

4 flies for each line were initially screened at 1000 lux of constant light. Since even control flies sometime show weak residual behavioral rhythms in LL, we selected only lines for which at least 2 flies were rhythmic, with a robust rhythm for at least one (i.e. a power over 20, see Ewer and al. [83] for power definition). Each selected line was then retested under constant light (LL) at least twice, usually 3 times, with a minimum of 8 flies per experiment.

#### Phase-responses

For each experiment, 16 flies per genotype were entrained for 3 full days in 12hr∶12hr LD. They were exposed to light (1000lux) at ZT15 or ZT21 (ZT0 corresponds to light on and ZT12 corresponds to light off) and returned in constant darkness for 5 full days. At the end of the experiment, all the flies that had survived the entire run and were scored as rhythmic using FaasX were analyzed. The phase was calculated for each group of flies in Matlab using the “peakphaseplot” function (the non-evening peaks were removed manually) as described in [18].

### Western blot

20 fly heads were homogenized in extraction buffer (20mM HEPES pH7.9, 100mM KCl, 5% Glycerol, 0.1% Triton X100, 0.1mM DTT and 1× protease inhibitor [Roche]), 5× SDS-PAGE loading buffer was then added, and samples denatured at 100°C for 10 minutes. After centrifugation, protein extracts were loaded on 5% and 6% 29.6∶0.4 acrylamide∶bisacrylamide gels for KIS and TIM, respectively, and a 9% 29∶1 acrylamide∶bisacrylamide gel (for Tubulin). Proteins were transferred on nitrocellulose and blots were incubated with primary antibody (1∶1000 for anti-KIS-L [43], 1∶5000 for anti-TIM [57] and 1∶10, 000 for anti-Tubulin [Sigma]) and HRP-conjugated secondary antibodies (Jackson Immuno Research). Films were imaged with the Fujifilm LAS-1000 and band intensities were quantified using the ImageGaugev4.22 software. KIS or TIM protein levels were normalized with Tubulin.

### Whole-mount brain immunostaining and quantification

Brains from adult flies were dissected in 1× PBS, 0.1% Triton (PBT), fixed in 4% paraformaldehyde in PBT and blocked in 10% Normal Goat Serum (Jackson Immuno Research) in PBT. They were then washed and incubated overnight at 4°C with primary antibodies. After several washes in PBT, they were incubated with secondary antibodies (Jackson Immuno Research) coupled to FITC, Rhodamine, Cy3 or Cy5 for 2–3hours at room temperature at a concentration of 1∶200. Brains were mounted in antifade reagent (Biorad or Vectashield).

The anti-KIS-L (generous gift from J. Tamkun) and anti-PDF antibodies were previously described [43],[85] and both used at a 1∶400 dilution. For ß-Gal immunostaining, we used a mouse anti-ß-Gal antibody (Promega) at a concentration of 1∶1000 as previously described in [58] and CRY antibody was used at a concentration of 1∶200. Levels of KIS protein expression in circadian neurons were quantified using ImageJ v1.42q (http://rsb.info.nih.gov/ij). For each genotype, 5 to 12 neurons from at least 3 different brains were quantified. For each circadian neuron, the nuclear fluorescence corresponding to KIS staining was measured and normalized, after subtraction of background signal, to the fluorescence of two neighbor neurons on the same focal slice. An average of the two normalized values was then calculated for each circadian neuron.

### Real-time quantitative PCR

Total RNAs from about 60 fly heads collected at ZT 4 and ZT16 were prepared using Trizol (Invitrogen) according to the manufacturer's instructions. 2 µg of total RNAs were then treated with RQ1 DNAse (Promega) for 2 hours and subsequently reverse transcribed using random hexamer primers (Promega) and Superscript II (Invitrogen), following manufacturer's instructions. Real-time PCR analysis was performed using SYBR Green fluorescent dye (Biorad) in an ABI SDS 7000 instrument (Applied Biosystems). For each set of primers, we generated a standard and a melting curve, using RNAs extracted from wild-type fly heads, to verify amplification efficiency and specificity, respectively. For each transcript, data were normalized to *rp32* using the 2^−ΔΔCt^ method. The concentration of *per*, *tim*, *cry*, *csn4*, *csn5*, *sgg* and *jet* were measured in flies expressing *kis* dsRNAs (*tim-GAL4 UAS-dcr2/VDRC46685*) and in two control genotypes (*tim-GAL4 UAS-dcr2/+* and *VDRC46685/+*). Primers used: *rp32*-forward ATGCTAAGCTGTCGCACAAA; *rp32*-reverse GTTCGATCCGTAACCGATGT; *tim*-forward TGAACGAGGACGACAAAGCC; *tim*-reverse GATTGAAACGCCTCAGCAGAAG; *per*-forward TCATCCAGAACGGTTGCTACG; *per*-reverse CCTGAAAGACGCGATGGTGT; *cry*-forward CCGCTGACCTACCAAATGTT; *cry*-reverse GGTGGCGTCTTCTAGTCGAG; *csn4*-forward AGCAAGTTGCCTGACGATCT; *csn4*-reverse GAAACGTATGCCAGCCACTT; *csn5*-forward ACCCAGATGCTCAACCAGAC; *csn5*-reverse CTTTTGGATACGTGCGGAAT; *sgg*-forward TGCTGCTCGAGTATACGCCC; *sgg*-reverse TCCATGCGTAGCTCATCGAAG; *jet*-forward CTGCTGCAGTCACTGATGGT; *jet*-reverse ATGTTGCACAGTTGGCATGT.

### RNase protections

RNase protections were performed and quantified with an *rp49* loading control as described in [17]. The *lk6* probe covered nucleotide 919–1055 of transcript *lk6*-RA. It generated two major bands. The larger one corresponds to transcript *lk6*-RA, and the smaller one most likely to *lk6*-RB. Quantification for the RA band is shown on Figure S4. The smaller band cycled with a similar phase and amplitude (data not shown).

## Supporting Information

Figure S1 Constant light phenotype of flies overexpressing genes involved in proteasomal degradation. UBCD1 (E2 ubiquitin conjuguase), AGO (F-box containing protein), and MORGUE (F-box containing protein with an E2 ubiquitin conjuguase domain) were overexpressed (O/E) by combining *tim-GAL4* with *UAS-ubcd1*, *EP(3)1135*, and *EP(2)2367*, respectively.
(1.25 MB TIF)

Figure S2 KIS protein levels are severely reduced in circadian neurons expressing *kis* dsRNAs. (A) KIS protein levels in large and small LNvs and in LNds measured by immunostaining. The histogram represents the normalized KIS fluorescence signal (see Materials and Methods for details), measured in control flies (*pdf-GAL4 UAS-dcr2*/+ [*pg4 dcr2*/+] and *tim-GAL4 UAS-dcr2; R32* [*tg4 dcr2*/+]) and flies expressing *kis* dsRNAs in all circadian neurons (*tim-GAL4 UAS-dcr2/VDRC46685; R32* [*tg4 dcr2/VDRC46685*]) or only in PDF positive neurons (*pdf-GAL4 UAS-dcr2/VDRC46685* [*pg4 dcr2/VDRC46685*]). KIS expression is reduced by about 85–90% in both mutant genotypes, and in all cell types surveyed. When driving *kis* dsRNAs with *pdf-GAL4*, we used an anti-PDF antibody to identify the PDF positive LNvs. With *tim-GAL4*, we used flies carrying one copy of the *R32 lacZ* insertion trap [58] and identified clock neurons with an anti-βGAL antibody. βGAL staining was weak in Dorsal Neurons, but a few DN1s could nevertheless be identified in *tg4 dcr2/VDRC46685* brains. KIS expression was also severely reduced in these neurons (data not shown). Error bars represent standard deviations. (B) Histogram showing the percentage of rhythmicity in constant light for control flies (*pdf-GAL4 UAS-dcr2*/+ [*pg4 dcr2*/+] and *tim-GAL4 UAS-dcr2*/+ [*tg4 dcr2*/+]) and flies expressing *kis* dsRNAs in all circadian neurons (*tim-GAL4 UAS-dcr2/VDRC46685* [*tg4 dcr2/VDRC46685*]; *ls-tim* homozygotes) or only in PDF positive neurons (*pdf-GAL4 UAS-dcr2/VDRC46685* [*pg4 dcr2/VDRC46685*]; *ls-tim* homozygotes) (n = 16 flies for each genotype). As also shown on Figure 5 with the NIG-Fly line, driving the VDRC *kis* dsRNAs only in PDF positive LNvs does not induce LL rhythmicity, even though as shown in (A) KIS expression is as efficiently repressed as with *tim-GAL4*. AR: complete arrhythmicity.
(0.51 MB TIF)

Figure S3 *cry* overexpression restores constant light arrhythmicity in flies expressing *kis* dsRNAs. (A) Actograms showing the locomotor activity of *tim-GAL4 VDRC46685*/+ (*tg4 VDRC46685*;+); and *tim-GAL4 VDRC46685/UAS-cry* flies (*tg4 VDRC46685;cry*) under LL conditions. Both genotypes are homozygous for *ls-tim* (n = 16 flies for each genotype). (B) Percentage of LL rhythmicity for the same genotypes.
(1.32 MB TIF)

Figure S4 *lk6* mRNA cycles in phase with *cry* mRNA. x axis: Zeitgeber Time, ZT (ZT0–12 = day, ZT12–24 = night). y axis: relative mRNA abundance measured by RNase protection.
(0.21 MB TIF)

Table S1 Behavior of the selected EP lines crossed to *tim-GAL4* under constant darkness (as = antisense orientation).
(0.07 MB DOC)

Table S2 Behavior of *EP(2)2356* crossed to different *GAL4* drivers and *GAL80* repressor transgenes under constant light (200 lux, unless otherwise indicted) (AR = arrhythmicity).
(0.03 MB DOC)

Table S3 Behavior of flies expressing *kismet* dsRNAs in constant darkness.
(0.04 MB DOC)
