# Supplementary figures and images for: A Constant Light-Genetic Screen Identifies KISMET as a Regulator of Circadian Photoresponses
Source: PLoS Genet. 2009 Dec 24;5(12):e1000787. doi: 10.1371/journal.pgen.1000787 (PMC2789323; doi:10.1371/journal.pgen.1000787)

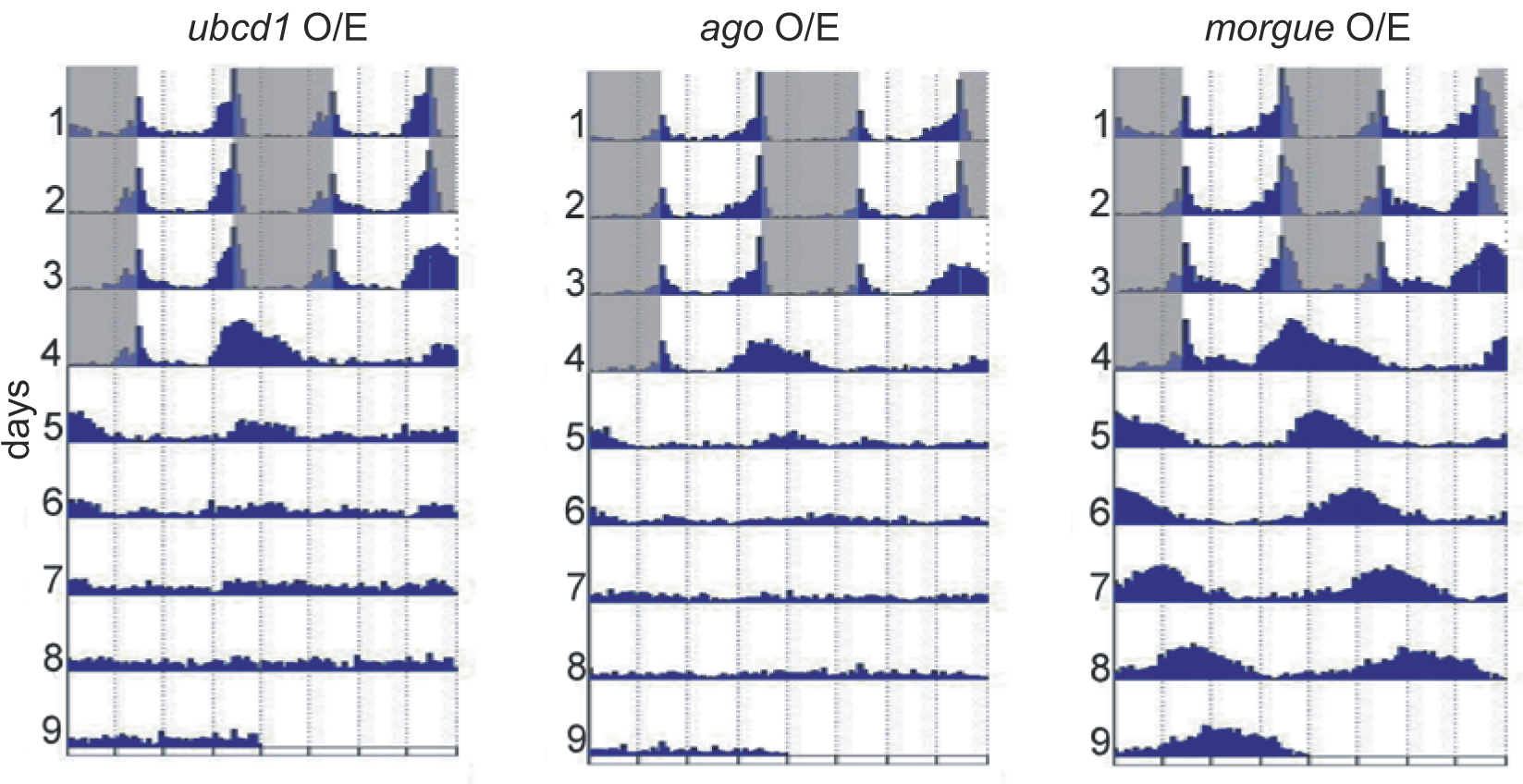

Supplement: Figure S1 — Constant light phenotype of flies overexpressing genes involved in proteasomal degradation. UBCD1 (E2 ubiquitin conjuguase), AGO (F-box containing protein), and MORGUE (F-box containing protein with an E2 ubiquitin conjuguase domain) were overexpressed (O/E) by combining tim-GAL4 with UAS-ubcd1, EP(3)1135, and EP(2)2367, respectively. (1.25 MB TIF) [file pgen.1000787.s001.tif]

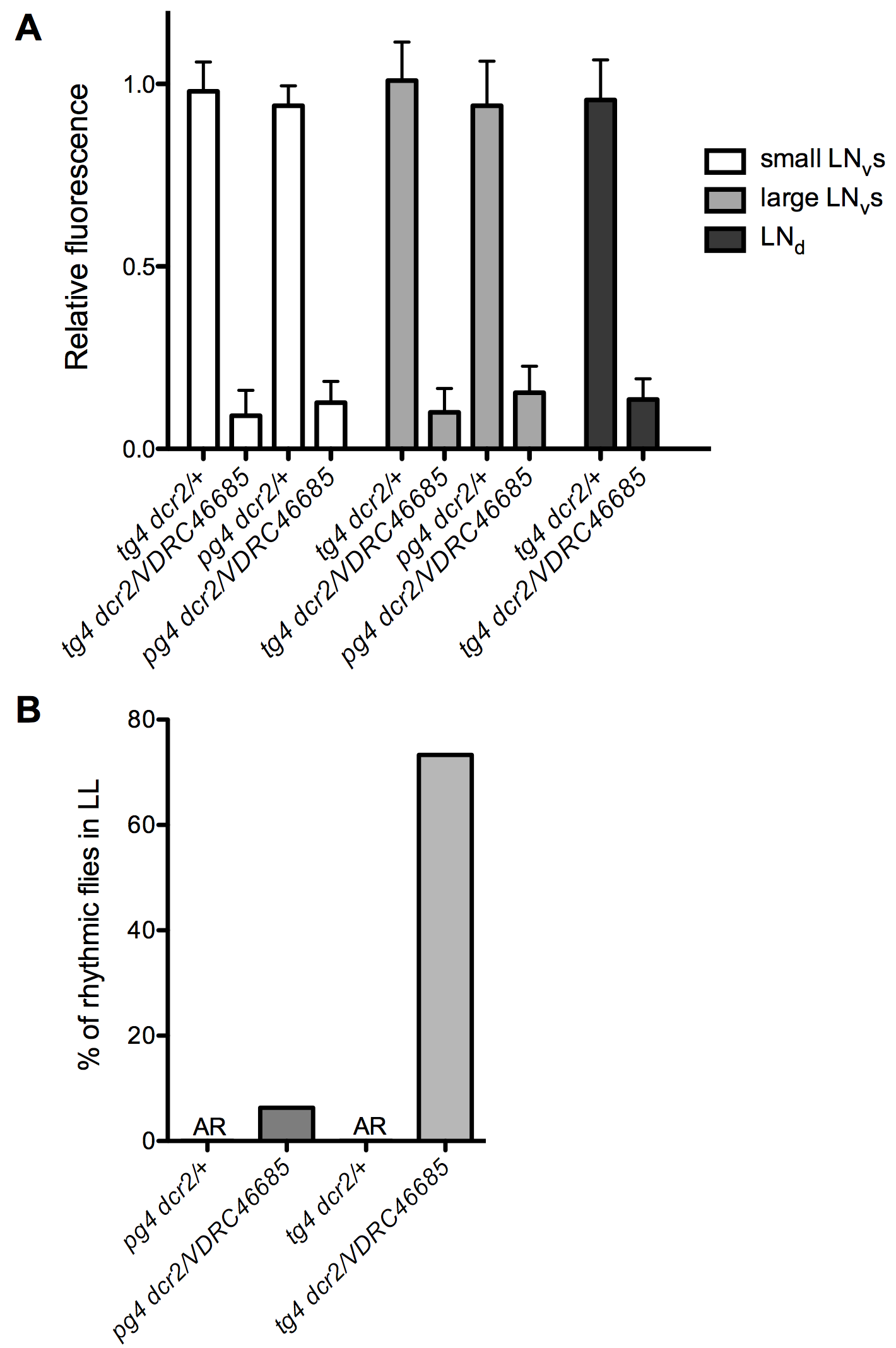

Supplement: Figure S2 — KIS protein levels are severely reduced in circadian neurons expressing kis dsRNAs. (A) KIS protein levels in large and small LNvs and in LNds measured by immunostaining. The histogram represents the normalized KIS fluorescence signal (see Materials and Methods for details), measured in control flies (pdf-GAL4 UAS-dcr2/+ [pg4 dcr2/+] and tim-GAL4 UAS-dcr2; R32 [tg4 dcr2/+]) and flies expressing kis dsRNAs in all circadian neurons (tim-GAL4 UAS-dcr2/VDRC46685; R32 [tg4 dcr2/VDRC46685]) or only in PDF positive neurons (pdf-GAL4 UAS-dcr2/VDRC46685 [pg4 dcr2/VDRC46685]). KIS expression is reduced by about 85–90% in both mutant genotypes, and in all cell types surveyed. When driving kis dsRNAs with pdf-GAL4, we used an anti-PDF antibody to identify the PDF positive LNvs. With tim-GAL4, we used flies carrying one copy of the R32 lacZ insertion trap [58] and identified clock neurons with an anti-βGAL antibody. βGAL staining was weak in Dorsal Neurons, but a few DN1s could nevertheless be identified in tg4 dcr2/VDRC46685 brains. KIS expression was also severely reduced in these neurons (data not shown). Error bars represent standard deviations. (B) Histogram showing the percentage of rhythmicity in constant light for control flies (pdf-GAL4 UAS-dcr2/+ [pg4 dcr2/+] and tim-GAL4 UAS-dcr2/+ [tg4 dcr2/+]) and flies expressing kis dsRNAs in all circadian neurons (tim-GAL4 UAS-dcr2/VDRC46685 [tg4 dcr2/VDRC46685]; ls-tim homozygotes) or only in PDF positive neurons (pdf-GAL4 UAS-dcr2/VDRC46685 [pg4 dcr2/VDRC46685]; ls-tim homozygotes) (n = 16 flies for each genotype). As also shown on Figure 5 with the NIG-Fly line, driving the VDRC kis dsRNAs only in PDF positive LNvs does not induce LL rhythmicity, even though as shown in (A) KIS expression is as efficiently repressed as with tim-GAL4. AR: complete arrhythmicity. (0.51 MB TIF) [file pgen.1000787.s002.tif]

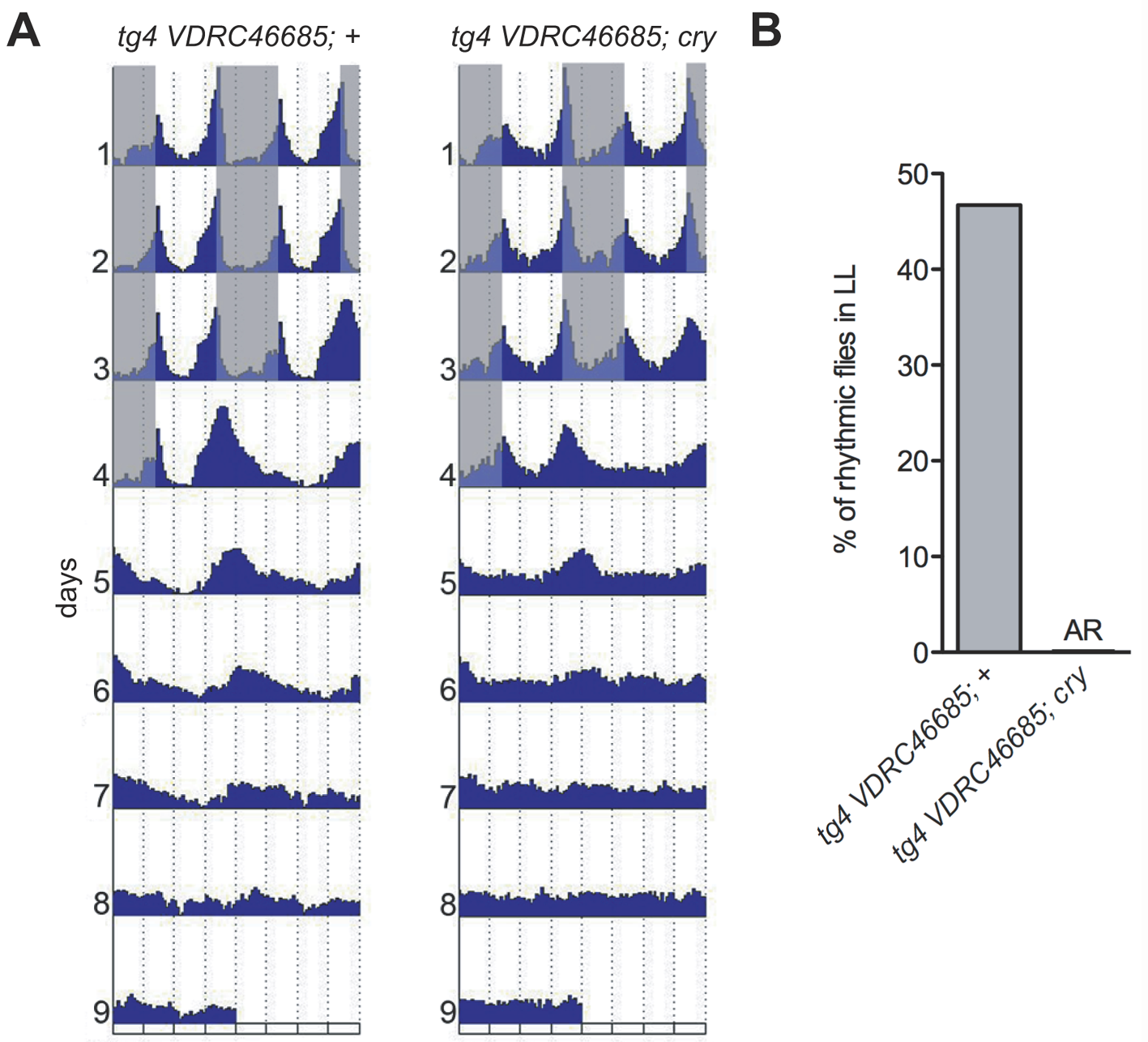

Supplement: Figure S3 — cry overexpression restores constant light arrhythmicity in flies expressing kis dsRNAs. (A) Actograms showing the locomotor activity of tim-GAL4 VDRC46685/+ (tg4 VDRC46685;+); and tim-GAL4 VDRC46685/UAS-cry flies (tg4 VDRC46685;cry) under LL conditions. Both genotypes are homozygous for ls-tim (n = 16 flies for each genotype). (B) Percentage of LL rhythmicity for the same genotypes. (1.32 MB TIF) [file pgen.1000787.s003.tif]

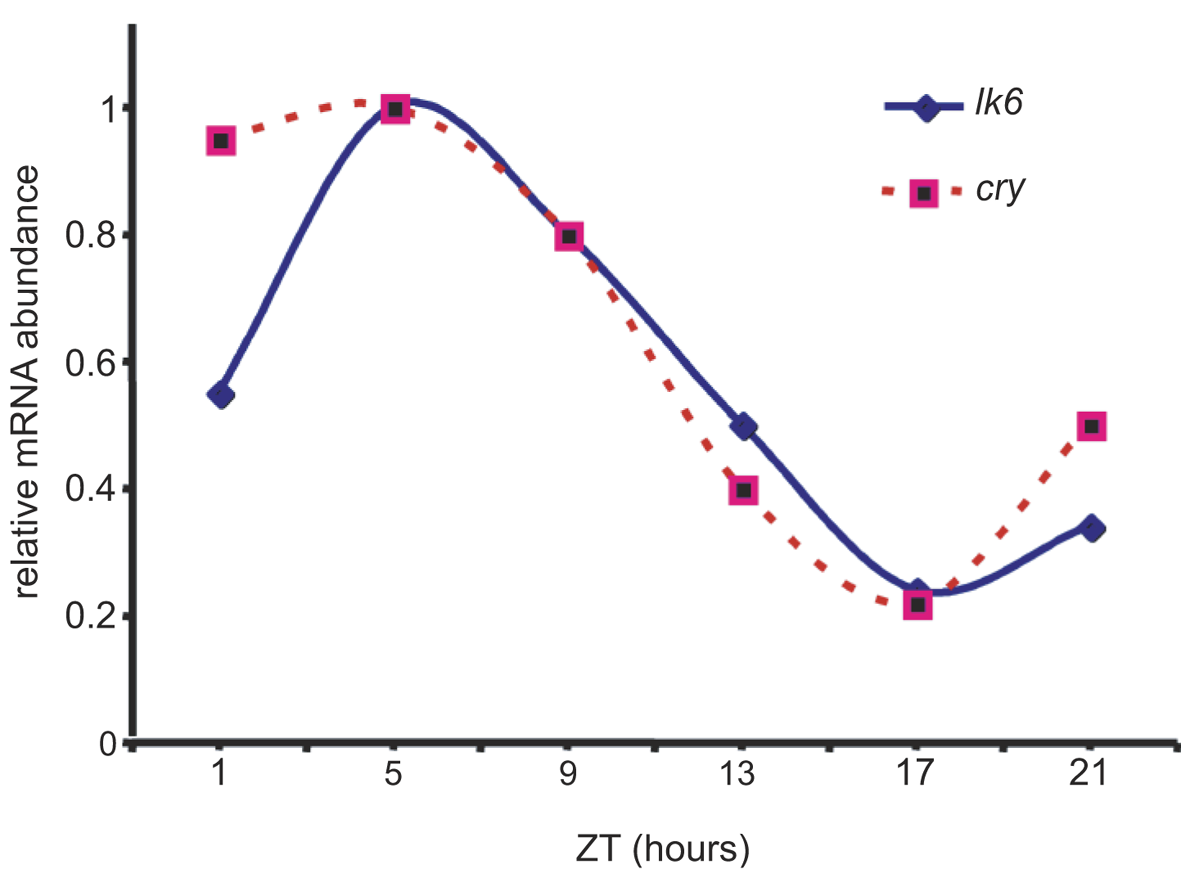

Supplement: Figure S4 — lk6 mRNA cycles in phase with cry mRNA. x axis: Zeitgeber Time, ZT (ZT0–12 = day, ZT12–24 = night). y axis: relative mRNA abundance measured by RNase protection. (0.21 MB TIF) [file pgen.1000787.s004.tif]
