# Supplementary material for: A Constant Light-Genetic Screen Identifies KISMET as a Regulator of Circadian Photoresponses
Source: PLoS Genet. 2009 Dec 24;5(12):e1000787. doi: 10.1371/journal.pgen.1000787 (PMC2789323; doi:10.1371/journal.pgen.1000787)
Supplement: Table S1 — Behavior of the selected EP lines crossed to tim-GAL4 under constant darkness (as = antisense orientation). (0.07 MB DOC) [file pgen.1000787.s005.doc]

| Genotype (each *EP* lines is crossed to *tim-GAL4*) | Gene predicted to be affected | n | % of rhythmic flies | Period average (± SD) | Power average  (± SD) |
| --- | --- | --- | --- | --- | --- |
| *EP(2)2367* | *morgue* | 12 | 83 | 24.5±0.3 | 72.9±27.2 |
| *EP(3)714* | *miR-282* | 11 | 55 | 24.9±0.4 | 59.6±35.6 |
| *EP(3)3718* | *miR-282* | 10 | 80 | 24.2±0.5 | 47.1±23.6 |
| *EP(2)670* | *GstS1* | 10 | 100 | 24.9±0.2 | 56.7±11.3 |
| *EP(3)703* | *cg8165/8176* | 22 | 100 | 24.8±0.4 | 54.2±21.7 |
| *EP(3)3041* | *miR-282* | 10 | 100 | 24.3±0.4 | 46.7±21.1 |
| *EP(2)965* | *elB* | 22 | 100 | 24.9±0.3 | 68.2±24.5 |
| *EP(3)972* | *calpB* | 11 | 55 | 25±0.3 | 33.3±10 |
| *EP(3) 902* | *kay* (as) or *cg1973* | 9 | 100 | 24.5±0.4 | 38.8±16 |
| *EP(3)614* | *cg12173* | 17 | 88 | 24.8±0.6 | 56.3±29 |
| *EP(2)506* | *HSPC300* | 19 | 95 | 24.8±0.4 | 62.8±26 |
| *EP(3)3617* | *miR-282* |  |  | ND |  |
| *EP(2)323* | *cg8735* | 19 | 95 | 24.6±0.2 | 60.3±26.6 |
| *EP(3)662* | *slimb* | 7 | 85 | 25.1±0.3 | 42.3±9.4 |
| *EP(2)2345* | *dap* (as) or *cg10459* (as) | 11 | 100 | 24.6±0.4 | 70.5±30.6 |
| *EP(3)3084* | *kay* | 15 | 93 | 24.7±0.3 | 59.6±39.5 |
| *EP(2)2319* | *cg10082* | 10 | 100 | 25.1±0.4 | 67.1±26.4 |
| *EP(2)575* | *Rapgap1 (as) or cg13791* (as) | 11 | 91 | 24.6±0.3 | 59.4±20.7 |
| *EP(2)813* | *wech* (as) or *cg1621* (as) | 10 | 80 | 24.5±0.3 | 59.3±26.9 |
| *EP(3)1141* | *sda* | 10 | 70 | 25±0.5 | 47.9±26.8 |
| *EP(3)1110* | *cg9801* | 12 | 100 | 24.7±0.4 | 44.8±20.7 |
| *EP(2)2241* | *Dg* | 19 | 100 | 24.7±0.3 | 71.8±24.3 |
| *EP(3)661* | *cpo* | 10 | 90 | 25±0.3 | 57.2±28.5 |
| *EP(2)2469* | *kis* (as) | 10 | 80 | 25±0.3 | 57.2±28.5 |
| *EP(2)2254* | *akap200* | 11 | 100 | 24.8±0.2 | 68.3±25.2 |
| *EP(3)996* | *cg31184/cg33108* | 11 | 64 | 25.1±1.4 | 33.6±12.6 |
| *EP(2)2356* | *miR-310/311/312//313* | 11 | 100 | 24.1±0.3 | 54.5±17.7 |
| *EP(2)2098* | *cg30152* | 11 | 100 | 24.6±0.3 | 81.8±35.9 |
| *EP(2)2402* | *miR-8* | 11 | 100 | 25.3±0.4 | 60±29 |
| *EP(3)3094* | *lk6* | 15 | 93 | 25.3±0.4 | 73.5±25.1 |
|  |  |  |  |  |  |
| Controls |  |  |  |  |  |
| *y w; tim-GAL4/+* |  | 12 | 100 | 24.8±0.4 | 52.9±18.8 |
| *cry^b^* |  | 12 | 75 | 23.9±0.4 | 84.7±40.4 |
