# Supplementary material for: A Constant Light-Genetic Screen Identifies KISMET as a Regulator of Circadian Photoresponses
Source: PLoS Genet. 2009 Dec 24;5(12):e1000787. doi: 10.1371/journal.pgen.1000787 (PMC2789323; doi:10.1371/journal.pgen.1000787)
Supplement: Table S2 — Behavior of EP(2)2356 crossed to different GAL4 drivers and GAL80 repressor transgenes under constant light (200 lux, unless otherwise indicted) (AR = arrhythmicity). (0.03 MB DOC) [file pgen.1000787.s006.doc]

| Drivers/  *EP(2)2356* | n | Short period  (τ<22 hr) | ca. 24hr (22<τ<26) | Long period (τ>26) | Short+long | Short + ca. 24hr | AR |
| --- | --- | --- | --- | --- | --- | --- | --- |
| *tim-GAL4* (1000lux) | 34 | 24% | 9% | 6% | 6% | 6% | 47 % |
| *tim-GAL4* | 37 | 22% | 8% | 5% | 18% | 3% | 43% |
| *tim-GAL4/*  *pdf-GAL80* | 40 | 20% | 5% | 5% | 41% | - | 28% |
| *tim-GAL4/*  *cry-GAL80* | 29 | 10% | 5% | 3% | - | - | 82% |
| *pdf-GAL4* | 32 | 12.% | - | - | - | - | 88% |
