# Supplementary material for: A Constant Light-Genetic Screen Identifies KISMET as a Regulator of Circadian Photoresponses
Source: PLoS Genet. 2009 Dec 24;5(12):e1000787. doi: 10.1371/journal.pgen.1000787 (PMC2789323; doi:10.1371/journal.pgen.1000787)
Supplement: Table S3 — Behavior of flies expressing kismet dsRNAs in constant darkness. (0.04 MB DOC) [file pgen.1000787.s007.doc]

| Genotype | % rhythmic flies | period ±SD | power±SD | n |
| --- | --- | --- | --- | --- |
| *y w* | 74.03% | 23.79±0.34 | 74.41±32.57 | 77 |
| *y w; cry^b^/cry^b^* | 72.58% | 24.13±0.43 | 79.86±42.03 | 62 |
| *y w; tim-GAL4/+* | 92.31% | 24.63±0.3 | 77.12±34.44 | 72 |
| *y w; NIG-Fly3696-R1/+* | 57.45% | 24.27±0.24 | 77.86±30.60 | 47 |
| *y w; tim-GAL4/+; NIG-Fly3696-R1/+* | 93.48% | 25.07±0.26 | 101.17±37.60 | 46 |
| *y w; VDRC46685/+* | 60.42% | 23.70±0.41 | 57.23±25.99 | 48 |
| *y w; tim-GAL4/VDRC46685* | 97.73% | 24.99±0.34 | 73.46±33.26 | 44 |
| *y w; tim-GAL4, UAS-dcr2/+* | 93.30% | 24.8±0.22 | 75.5± 19.31 | 15 |
| *y w; tim-GAL4, UAS-dcr2/VDRC46685* | 68.80% | 25.4±0.23 | 54.2± 20.1 | 16 |
